# Impact of Industrially Affected Soil on Humans: A Soil-Human and Soil-Plant-Human Exposure Assessment

**DOI:** 10.3390/toxics10070347

**Published:** 2022-06-22

**Authors:** Yeasmin N. Jolly, Md. Refat Jahan Rakib, M. Sadman Sakib, M. Ashemus Shahadat, Arafat Rahman, Shirin Akter, Jamiul Kabir, M. Safiur Rahman, Bilkis A. Begum, Rubina Rahman, Abdelmoneim Sulieman, Nissren Tamam, Mayeen Uddin Khandaker, Abubakr M. Idris

**Affiliations:** 1 Atmospheric and Environmental Chemistry Laboratory, Atomic Energy Centre, P.O. Box 164, Dhaka 1000, Bangladesh; shirinakhter43@yahoo.com (S.A.); jamiulkabir@gmail.com (J.K.); safiur_baec@yahoo.com (M.S.R.); bilkisab@dhaka.net (B.A.B.); 2 Department of Fisheries and Marine Science, Noakhali Science and Technology University, Noakhali 3814, Bangladesh; 3 Department of Physics, Jahangirnagar University, Savar, Dhaka 1342, Bangladesh; sadmansakib428@gmail.com (M.S.S.); shahadatsulov@gmail.com (M.A.S.); r.rahman@Liverpool.ac.uk (R.R.); 4 Department of Soil, Water and Environment, University of Dhaka, Dhaka 1000, Bangladesh; arafat.du.edu@gmail.com; 5 Department of Radiology and Medical Imaging, College of Applied Medical Sciences, Prince Sattam bin Abdulaziz University, P.O. Box 422, Alkharj 11942, Saudi Arabia; a.sulieman@psau.edu.sa; 6 Department of Physics, College of Science, Princess Nourah bint Abdulrahman University, P.O. Box 84428, Riyadh 11671, Saudi Arabia; nmtamam@pnu.edu.sa; 7 Centre for Applied Physics and Radiation Technologies, School of Engineering and Technology, Sunway University, Bandar Sunway 47500, Selangor, Malaysia; mu_khandaker@yahoo.com; 8 Department of General Educational Development, Faculty of Science and Information Technology, Daffodil International University, DIU Rd, Dhaka 1341, Bangladesh; 9 Department of Chemistry, College of Science, King Khalid University, Abha 62529, Saudi Arabia; abubakridris@hotmail.com; 10 Research Center for Advanced Materials Science (RCAMS), King Khalid University, Abha 62529, Saudi Arabia

**Keywords:** health risk, metal translocation, multivariate statistical analysis, pollution degree, soil, vegetables

## Abstract

Heavy metal (HM) contaminated soil can affect human health via ingestion of foodstuffs, inhalation of soil dust, and skin contact of soil. This study estimates the level of some heavy metals in soils of industrial areas, and their exposures to human body via dietary intake of vegetables and other pathways. Mean concentrations of Cr, Fe, Cu, Zn, As and Pb in the studied soil were found to be 61.27, 27,274, 42.36, 9.77, 28.08 and 13.69 mg/kg, respectively, while in vegetables the respective values were 0.53, 119.59, 9.76, 7.14, 1.34 and 2.69 mg/kg. Multivariate statistical analysis revealed that Fe, Cu, Zn, and Pb originated from lithogenic sources, while Cr and As are derived from anthropogenic sources. A moderate enrichment was noted by Cr, As, and Pb in the entire sampling site, indicating a progressive depletion of soil quality. The bioaccumulation factor (BCF) value for all the vegetables was recorded as BCF < 1; however, the metal pollution index (MPI) stipulates moderately high value of heavy metal accumulation in the vegetable samples. Hazard Index (HI) of >0.1 was estimated for adults but >1 for children by direct soil exposure, whereas HI < 1 for both children and adults via dietary intake of vegetables. Estimated Total carcinogenic risk (TCR) value due to soil exposure showed safe for adults but unsafe for children, while both the population groups were found to be safe via food consumption. Children are found more vulnerable receptors than adults, and health risks (carcinogenic and non-carcinogenic) via direct soil exposure proved unsafe. Overall, this study can be used as a reference for similar types of studies to evaluate heavy metal contaminated soil impact on the population of Bangladesh and other countries as well.

## 1. Introduction

The fundamental part of the ecosystem is the soil which supplies necessary nutrients to living organisms. Soil receives different types of metals (heavy metals, toxic and trace elements) from various sources (anthropogenic and lithogenic), however, an increase of their natural content may reduce the soil quality. In fact, as a reservoir, the soil itself is abundant in metals transported from biomass, atmosphere, and hydrosphere [1,2], but it may pose a risk to human health and ecosystem when exceeded the safe/threshold limit. Due to non-degradable characteristics and toxicity, contamination of soil by heavy metal has caught the major concern worldwide. Among the various anthropogenic sources: mining, smelting, industrialisation, agrochemicals, urbanisation, domestic wastes, and transportation are the major contributor, while for lithogenic input, weathering and erosion of bedrocks and ore deposit etc. have come to the forefront [3,4,5]. Some heavy metals (Cu, Zn, Fe, Cr, Mn) are essential and some are toxic (As, Pb, Cd, Hg) for human health but exposure to a level more than acceptable range cause deleterious health hazard. Pb, Cr, Cu, Zn, As are usually accumulated in the fatty tissues of human body; Pb is responsible for disruption of normal organ function, and affect the nervous system; As, Cr and Pb are considered as carcinogenic elements, which are responsible for various type of cancer in the human body [6,7,8]. 

Heavy metal (HM) contaminated soil may pose potential risks and hazards to humans by direct ingestion or contact with contaminated soil or inhalation of contaminated soil dust and intake of foodstuffs. Thus, health risks arising from soil can be estimated by calculating various soil pollution indices of HMs, their soil-to-plant transfer factors, direct exposure level to humans, and their levels in edible food crops as well as health risk due to consumption of contaminated food crops. Numerous studies have been conducted all over the world and so as in Bangladesh, to estimate the health hazards of heavy metal contaminated soil [9,10,11,12,13] and food contamination [14,15,16,17,18,19,20] individually. However, in this study health effects due to heavy metal contaminated soil and vegetables grown on the same industrially affected soil have been computed. Moreover, to ascertain the degree of pollution, soil from a non-contaminated area having a similar soil texture was also analysed to get the baseline soil data of that particular area.

Ashulia, a neighbouring community of Dhaka district (the capital of Bangladesh), which is a suburban area, and Savar a nearby area having the same soil texture were targeted as the sampling area. A vast number of paddy fields and agricultural lands are located in this area. Ashulia Lake and two major theme parks in Bangladesh, namely “Fantasy Kingdom” and “Nandan Park”, make it a popular tourist location. A huge number of local and foreign tourists including children visited this area frequently. However, in recent years, rapid urbanization, the establishment of garments factories, bricks fields and other factories deteriorated its beauty and reduced the farmlands. Frequent dumping of untreated solid and liquid wastes from the factories and brickfields to the nearby agricultural land makes them assailable due to the risk of metal accumulation into crops, vegetables and ultimately the human body. As far as our concern, no studies have been conducted to monitor this industrially affected land soil, crops grown in these soils and their impact on humans. In this context, the present study was aimed to measure some essential and non-essential metals (Fe, Cr, Cu, Zn, As, Pb) in soil contaminated by industrial wastes, and vegetables grown on this soil, to determine the degree of pollution by estimating various indices, and to find out the possible pollution sources by employing multivariate statistical analysis. Health risk owing to soil-human and the soil-plant-human route was assessed and a comparison was made to ascertain which path is more vulnerable. Translocation of heavy metals from soil to edible parts of vegetables was also carried out to find out the metal extraction capability of the plants from a phytoremediation point of view.

## 2. Material and Methods

### 2.1. The Study Site and Sample Collection

**Figure 1:**
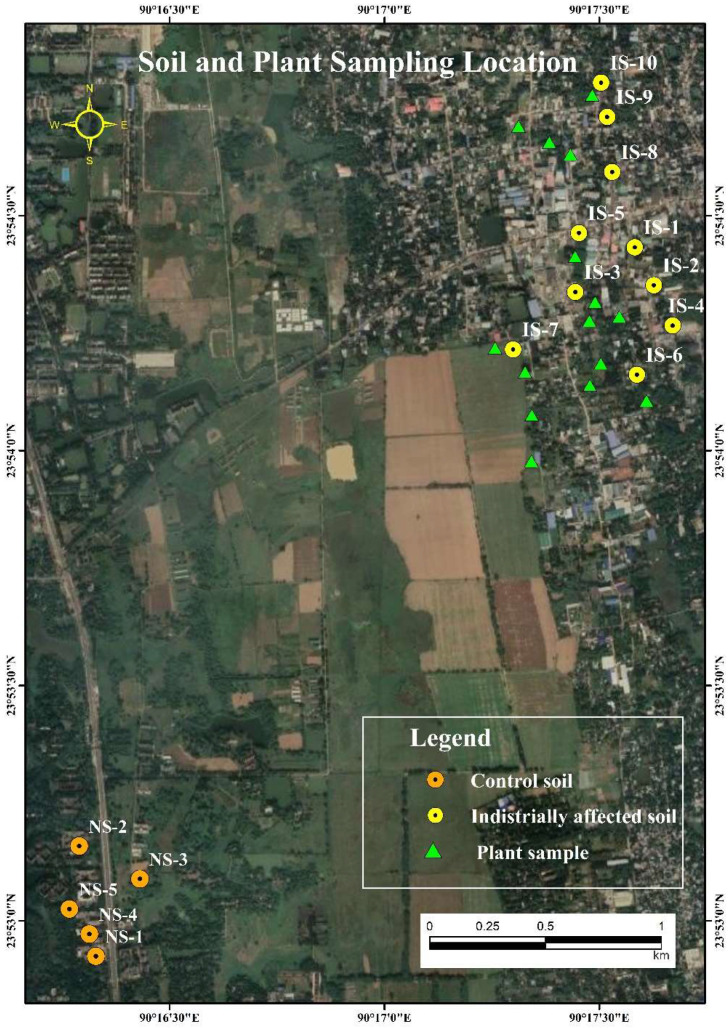
Map of soil (control and affected) and vegetable sampling locations.

Ten industrially affected soil samples designated as IS1 (23°54′19.1″ N 90°17′35.0″ E), IS2 (23°54′19.9″ N 90°17′36.3″ E), IS3 (23°54′16.3″ N 90°17′38.2″ E), IS4 (23°54′15.9″ N 90°17′38.4″ E), IS5 (23°54′16.1″ N 90°17′39.8″ E), IS6 (23°54′12.4″ N 90°17′37.3″ E), IS7 (23°54′12.9″ N 90°17′18.0″ E), IS8 (23°54′35.6″ N 90°17′31.8″ E), IS9 (23°54′42.6″ N 90°17′31.1″ E) and IS10 (23°54′47.0″ N 90°17′30.3″ E) were collected from the upper surface region (5–15 cm depth) of agricultural land of Ashulia, Dhaka (Figure 1). A large number of industrial establishments comprised of local and foreign industries such as fabric printing and dyeing, food processing, textiles, electric cables, pharmaceutical, chemical, etc., are located near the sampling station and wastes from those industries are dumped regularly. In addition, five soil samples Ns1 (23°52′49.4″ N 90°15′38.1″ E), Ns2 (23°52′49.6″ N 90°15′36.8″ E), Ns3 (23°52′53.1″ N 90°15′39.0″ E), Ns4 (23°52′49.3″ N 90°15′42.8″ E) and Ns5 (23°52′54.6″ N 90°15′38.6″ E), having the equal soil texture, considered as control soil and used as background soil were collected from Jahangirnagar University, Savar (Figure 1)**,** where industrial input was observed to be absent. To evaluate contaminated soil impact on human health via food consumption, 15 varieties of vegetables namely Spinach, Cabbage, Red Amaranth, Coriander leaf, Tomato, Brinjal, Bean, Pumpkin, Bottle gourd, Papaya, Green banana, Cauliflower, Carrot, Radish, Potato was collected that have been grown in and around the industrially affected soil sampling sites (Figure 1).

### 2.2. Sample Preparation

Each soil sample was dried to remove moisture, ground to a fine powder and finally sieved to obtain homogeneous particles. Vegetable samples were initially cleaned with tap water and rinsed with deionized water to remove any trace of soil and to minimize ion content, cut into small pieces, and dried to remove moisture. The dried mass was ground to a fine powder. Finally, 0.1 gm of each soil and vegetable sample in triplicate were pressed into a pellet of 0.7 cm diameter and 1 mm thickness using a pellet maker. The whole process is outlined by following the procedure described elsewhere [21,22].

### 2.3. Elemental Analysis of Soil and Vegetable Sample Using EDXRF

Soil and vegetable samples, each in triplicates, were analysed for heavy metals (Cr, Fe, Cu, Zn, As, Pb) using Energy Dispersive X-ray Fluorescence (EDXRF) system. It’s a non-destructive and multi-elemental analytical technique, well suited for solid sample (soil, sediment, vegetables, foodstuff, etc.) analysis. Many research works have been conducted with this technique for heavy/trace metal determination in environmental and food samples [15,17,22,23]. As sample preparation is very simple, no chemical treatment or digestion is required, thus reducing system loss of any sample mass [24], moreover, the accuracy and precision of the obtained data remain excellent. The quality assurance and quality control (QA/QC) of the soil and vegetable data were addressed by using certified reference materials (Montana-1/2710a for soil and Orchard leaf/NIST 15,710 for vegetable samples), where the recovery percentage of heavy metals (HMs) in the samples ranged from 93% to 106%, and the relative error for standard reference materials was around 5%. The entire process was described elsewhere [7,21].

### 2.4. Determination of HMs Contamination Status through Indices for Soil

The degree of soil pollution was measured by calculating the Enrichment factor (EF), Geo-accumulation index (I_geo_), Contamination factor (CF), and Pollution load index (PLI) as per [25], whereas plant contamination levels were calculated by using Bioaccumulation Factor (BCFs) and Metal pollution index (MPI) as per [15,23]. The equation used to calculate the contamination indices are:

(1) $$\mathrm{EF}=\frac{{\left(\frac{\mathrm{Me}}{\mathrm{Fe}}\right)}_{\mathrm{sample}}}{{\left(\frac{\mathrm{Me}}{\mathrm{Fe}}\right)}_{\mathrm{background}}}$$

where, EF refers to enrichment factor, (Me/Fe)_sample_ refers to the ratio of concentration between the studied metal and Fe in the sample of interest; (Me/Fe)_background_ is the natural background value (control soil in this case) of measured metal to Fe ratio [26]. However, EF lies in the classes as EF = 1, crustal materials or natural weathering processes, EF < 2 (Deficiency to minimal enrichment), 2 ≤ EF < 5 (Moderate enrichment), 5 ≤ EF < 20 (Significant enrichment), 20 ≤ EF < 40 (Very high enrichment) and EF ≥ 40 (Extremely high enrichment).

(2) $$I_{\mathrm{geo}}=\mathrm{Log}\;2\times \frac{C_{n}}{1.5B_{n}}$$

where, I_geo_ is the geo-accumulation index; C_n_ is the individual heavy metal concentration; B_n_ is the geochemical background value (Control soil value) and factor 1.5 is introduced to include possible variations of the background values due to the lithogenic effect [27]. I_geo_ value can be categorised [28] as I_geo_ ≤ 0 (unpolluted), I_geo_ = 0–1 (unpolluted to moderately polluted), I_geo_ = 1–2 (moderately polluted), I_geo_ = 2–3 (moderately to strongly polluted), I_geo_ = 3–4 (strongly polluted), I_geo_ = 4–5 (strongly to extremely polluted) and I_geo_ = 5–6 (extremely polluted).

(3) $$\mathrm{CF}=\frac{{\mathrm{Cm}}_{\mathrm{sample}}}{{\mathrm{Cm}}_{\mathrm{background}}}$$

where, CF is the contamination factor; C_m_sample__ is the concentration of a given metal; C_m_background__ is the background value of the metal (control soil) [29]. CF is categorised [28] as CF < 1 (low contamination), 1 ≤ CF < 3 (moderate contamination), 3 ≤ CF < 6 (considerable contamination) and CF ≥ 6 (very high contamination).

PLI = (CF_1_ × CF_2_ × CF_3_ ×CF_n_)^1/n^ (4)

where, PLI is the pollution load index; n is the number of metals to be analysed and PLI is categorised by [29] as PLI < 1 denotes perfection; PLI = 1 denotes baseline levels pollutants; PLI > 1 indicates deterioration of site quality.

### 2.5. Apportionment of Possible Sources of Soil Pollution

Multivariate statistical methods are usually applied to evaluate the complex eco-toxicological processes regarding the relationship and interdependency among the variables and their relative weights [30]. In this study, a popular multivariate statistical method, principal component analysis (PCA) was employed to verify the significant relationships between various heavy metals in the soil samples [31], and the cluster analysis (CA) was carried out to characterize notable variability among sites, using Euclidean distance for dissimilarity matrix and Ward’s method as the linkage method [32]. Ward’s method defines the proximity between two clusters as the increase in the squared error.

The data obtained from this study were analysed statistically using SPSS version 25.0 software (IBM SPSS Inc., USA), graphs were displayed using Microsoft Excel 2019, and Box-whisker plots were plotted with Origin Pro software version 9.0. The analysis of variance (ANOVA) tests at a significance level of 95% were used to evaluate the impact of different variables on the contamination in the study area. PCA and CA were performed using SPSS.

### 2.6. Determination of HMs Contamination Status through Indices for Vegetables

Vegetable contamination levels were calculated by using Bioaccumulation Factor (BCFs) and Metal pollution index (MPI) as per [15,23].

The Equation (5) is used to calculate bioaccumulation factors (BCFs) of the heavy metals from soil to plant,

(5) $$\mathrm{BCFs}=\frac{C_{\mathrm{veg}}}{C_{\mathrm{soil}}}$$

where, C_veg_ is the concentration of heavy metal in the vegetable (mg/kg, dw), and C_soil_ is the concentration of heavy metal in the soil (mg/kg, dw) [33]. It is notable that the translocation abilities of the heavy metals from soil to the edible parts of the vegetables can be evaluated by this factor, and BCF > 1 reveals the plant can effectively translocate heavy metals from soil to the edible portion of the vegetables [23].

The Metal Pollution Index (MPI) index was obtained by calculating the geometrical mean concentration of all the metals in the analysed vegetable samples [21,34].

(6) $$\mathrm{MPI}\;\left(\mathrm{mg}/\mathrm{kg}\right)=\sqrt[{n}]{\mathrm{Cf}1\times \mathrm{Cf}2\times \mathrm{Cfn})}$$

where, Cf_n_ is the concentration of n number of metals in the sample.

### 2.7. Human Exposure and Health Risk Assessment Indices

Heavy metal contaminated soil can affect human health in two pathways: (1) soil to humans via direct soil (dust) exposure; (2) soil to food to humans via consumption of foodstuffs.

#### 2.7.1. Soil to Human Health Risk Assessment

Ingestion of particles (ADD_ing_); inhalation (ADD_inh_); dermal absorption of metals via the skin (ADD_Dermal_) [35] are the three main routes for direct exposure of soil to humans and are evaluated by the equation suggested in [36,37]. Thus, the non-carcinogenic risk, Hazard Quotient (HQ) for heavy metal contaminated soil was measured by using the Equation (7):

(7) $$\mathrm{HQ}=\frac{\mathrm{ADD}\;}{\mathrm{RfD}}$$

where, ADD refers to the dose due to the exposure of heavy metals (ADD_inges_ + ADD_inha_ + ADD_derm_) and RfD refers to the heavy metal (HM) oral reference dose. RfD for ingestion: Fe = 7.00 × 10^−^^1^, Cr = 3.00 × 10^−^^3^, Cu = 0.04, Zn = 0.3, As =3.00 × 10^−^^4^ and Pb = 0.0035; for inhalation: Cr =2.86 × 10^−^^5^, Cu = 0.0402, Zn = 0.3, As = 3.01 × 10^−^^4^ and Pb = 0.00352; for dermal contact: Cr = 6.00 × 10^−^^5^, Cu = 0.012, Zn = 0.06, As = 1.23 × 10^−^^4^ and Pb = 0.000525 [35,38,39,40,41].

The non-carcinogenic effect for n number of heavy metals, on the population, is the sum of all HQs, represented as the Hazard Index (HI), [36]. Hence, it is worthy to mention that HI < 1 denotes highly unlikely significant toxic interaction and HI > 1 denotes potential non-cancer health effect [42].

On the other hand, carcinogenic risks (CR) are estimated by the Equation (8):

CR = LAAD × SF(8)

where, LAAD = (LAAD_ing_ + LAAD_inh_ + LAAD_derm_) is the lifetime average daily dose expressed as a weighted average for each exposure path, SF is the slope factor for a particular carcinogenic element [37,40,43]. SF value for ingestion, As = 1.5, Pb = 0.009; for inhalation, As = 1.51, Cr = 4.20 × 10; for dermal, As = 3.66, Cr = 2.00 × 10 [40]. Notably, the value within the range of 1.0 × 10^−^^4^ to 1.0 × 10^−^^6^ is considered an acceptable level [36] but when the value exceeds 1.0 × 10^−^^4^ then, it is considered a lifetime carcinogenic risk to the person exposed. Detailed indices (non-carcinogenic and carcinogenic) are computed in Table 1. Li et al. [44] and Orosun [45] suggested seven categories of risk due to exposure of carcinogenic metal:< 1 × 10^−^^6^ (level I, extremely low risk); 1 × 10^−^^6^ to 1 × 10^−^^5^ (level II, low risk); 1 × 10^−^^5^ to 5 × 10^−^^5^ (level III, low-medium risk); 5 × 10^−^^5^ to 1 × 10^−^^4^ (level IV, medium risk); 1 × 10^−^^4^ to 5 × 10^−^^4^ (level V, medium to high risk); 5 × 10^−^^4^ to 1 × 10^−^^3^ (level VI, high risk); >1 × 10^−^^3^ (level VII, extremely high risk).

#### 2.7.2. Soil to Food to Human Health Risk Assessment

Estimated Daily Intake (EDI) of metals, Target Hazard Quotient (THQ), Hazard Index (HI), Cancer Risk (CR) and Total Cancer risk (TCR) are the indices addressed to estimate probabilistic risk due to consumption of vegetables grown in contaminated soil and detailed of the indices are computed in Table 1 and Table 2.

## 3. Results and Discussion

### 3.1. Heavy Metal Contents in Soil Samples

The concentration of heavy metals (Fe, Cr, Cu, Zn, As, Pb) in the industrially affected soil along with control soil is presented in Table 3. The ranges of the heavy metal in the affected soil are 68.19–51.18, 34,900–21,840, 51.78–32.24, 57.94–44.88, 37.34–18.19, 18.53–9.03 mg/kg for Cr, Fe, Cu, Zn, As, Pb, respectively. The mean value of Cr, Fe, Cu, Zn, As and Pb in control soils is 13.4, 21,570, 32.43, 35.33, 6.03, and 5.61 mg/kg, respectively (Table 3). Compared to control soil, affected soil ascertained a higher value (Table 3), however, the mean value of industrially affected soil can be ranked as Fe > Cr > Zn > Cu > As > Pb. According to the “World soil average” reported by Kabita-Pendias [47], the value of Cr, Zn, Cu, As and Pb are 59.5, 70.0, 38.9, 6.83 and 27.0 mg/kg, respectively, and hence the measured value in the present study is higher with an exception for Zn and Pb (Table 3). Jiang et al. [48] and Toth et al. [49] believed that the soil of old and more industrialized areas is comparatively high in elemental concentration. However, Antoniadisa et al. [50] reported mean concentrations of Fe, Cr, Cu, Zn, As, Pb as 31,488, 438.29, 39.78, 69.23, 100.33, 2.45 mg/kg, respectively, in soil samples of an industrial area of Volos, Greece which were higher than the present value except for Pb. Meanwhile, Rahman et al. [40] reported a consistent mean concentrations of Fe, Cu, Zn, and Pb were 21,163, 40.2, 77.0 and 19.5 mg/kg, respectively, in the topsoil samples collected from schools of different locations in Dhaka city, Bangladesh. Furthermore, Jolly et al. [21] also reported mean concentrations of Fe, Cr, Cu, Zn, As and Pb were 34,500, 58, 53, 98, 41 and 15 mg/kg, respectively, in the surface soil of Ishwardi, Pabna, Bangladesh, which was higher than the present study except for Cr. Nevertheless, Gupta et al. [12] observed concentrations of Zn, Pb, Cu and Co as 44.43, 14.62, 14.66 and 8.96 mg/kg in the agricultural soil sample of North India, which are almost consistent with the present findings.

### 3.2. Evaluation of Pollution Level in the Studied Soil

Environmental ecological risk by the HMs (Cr, Fe, Cu, Zn, As, Pb) was assessed by calculating single indices such as, enrichment factor (EF), geo-accumulation index (I_geo_), contamination factor (CF) and Pollution load index (PLI), and measured values are computed in Table 4. Measured EF value ranges of 2.80–4.754, 0.823–1.491, 0.795–1.446, 1.646–4.957, 0.995–3.262 for Cr, Cu, Zn, As and Pb among the sites, respectively (Table 4). According to Mohammad et al. [51], when EF < 1.5, the elements are most likely earth’s cluster origin, resulting from natural processes. In this study, Fe showed enrichment factor 1 for all the sites, indicating cluster metal, coming from weathering practice [52]. Cr and As were found to show moderate enrichment (2 ≤ EF < 5) for all the sites, indicating anthropogenic impact [26], while Pb showed miscellaneous enrichment values (Table 4) among the sites of the study area, indicating both cluster and anthropogenic origin. Furthermore, Cu and Zn showed enrichment < 2 for all sites, indicating deficiency to minimal enrichment and of geological origin. According to Zhang et al. [53], ranges of EF values were 1.10–10.95, 4.45–18.95, 0.71–2.77, 0.76–1.67, 0.73–2.28, 0.55–2.09 and 0.80–2.09 for As, Cd, Cr, Cu, Ni, Pb and Zn, respectively, in the soils along a wetland-forming Chrono sequence in the Yellow River Delta of China, which are almost similar with the EF value of present study, with an exception of As. Rahman et al. [40] also reported the average EF values of Cu, Zn, As, Pb were 1.96, 1.29, 2.98, 1.23, respectively, in the soils of the Dhaka city schools, Bangladesh, indicating moderate enrichment, which agrees with the present study.

The assessment of heavy metal contamination in soil based on the geochemical background of the metal can be calculated by evaluating I_geo_ value [54]. This study calculated I_geo_ for Fe, Cr, Cu, Zn, As and Pb, and it was found to vary from element to element. The result revealed I_geo_ = 0–1 for Fe for the sites IS6 and IS10 indicating unpolluted to moderately polluted by Fe, but in all other sites, I_geo_ < 0 for Fe (Table 4), indicating minimal anthropogenic effects and recommended unpolluted by Fe. In the case of Cu and Zn, I_geo_ = 0–1 was found in the site IS6, IS7, IS10 and IS6, IS8, IS9, respectively, indicating unpolluted to moderately polluted status by the elements. At the same time, I_geo_ < 0 was measured in the sites IS1, IS2, IS3, IS4, IS5, IS8, IS9 and IS1, IS2, IS3, IS4, IS5, IS7, IS10, for Cu and Zn, respectively, stipulating no pollution. In contrast, Cr, As and Pb showed I_geo_ = 0–1 for all the soil samples, recommended unpolluted to moderately polluted by Cr, As and Pb. In a previous study [40], I_geo_ value for different soil samples of Dhaka city of Bangladesh was found −0.41 to 0.68, 0.77 to 1.68, −0.47 to 1.14, 1.52 to 2.02, −0.64 to 0.75, 2.91 to 4.13, −0.03 to 0.85, −1.37 to 0.27, −0.33 to 1.16, −4.03 to 0.08, and −1.93 to 0.90 for Fe, Cu, Zn, As, Pb, Ti, Rb, Sr, Zr, K and Ca, respectively, which are almost similar to the present findings. However, Negahban et al. [22] reported I_geo_ were 1.20–0.57, 1.32–0.98, 2.97–0.88 and 1.26–0.58 for Cu, Zn, Pb, and Cd, respectively, in soils of a large alluvial fan located in Neyriz, Iran, which is higher than the present study and the possible reason may be different soil texture.

The contamination factors (CF) of the studied HMs are summarized in Table 4, which revealed all the sites are considerably contaminated by Cr (3.805–5.070); considerable to very highly contaminated by As (3.017–6.192), moderately contaminated by Fe (1.060–1.618), Cu (0.994–1.597), Zn (1.270–1.640) and Pb (1.610–3.303) but somehow in some sites (IS2, IS4, IS7) Pb showed the CF value 3 ≤ CF < 6 and hence appraising considerable contamination. Prosad et al. [55] also estimated considerable contamination by Pb, low- moderate contamination by Ni and As, and low-moderate-considerable contamination by Cu and Pb in agricultural soil of Daulatpur, Kushtia district, Bangladesh. However, Zabir et al. [56] reported a higher level of CF value (CF > 5) compared to the present study for Pb, Rb, Mg and Zn in soil samples adjacent to the Bhaluka Industrial Area, Mymensingh district, Bangladesh.

**Figure 2:**
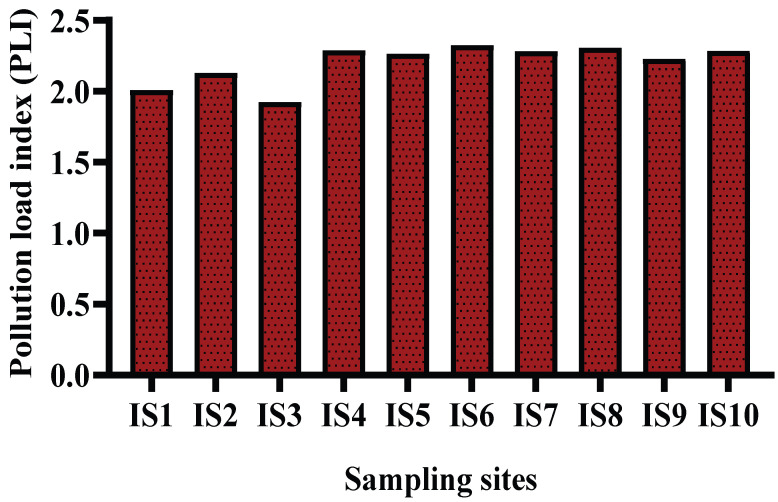
Pollution load index of the sampling sites of the study area.

The pollution load index (PLI) was calculated to assess the integrated index of pollution by heavy metals in the contaminated soil, which is depicted in Figure 2. PLI values were observed in the decreasing order of IS6 (2.326) > IS8(2.307) > IS4(2.291) > IS10(2.287) > IS7(2.285) > IS5(2.265) > IS9(2.231) > IS2(2.130) > IS1(2.009) > IS3 (1.927) and found PLI > 1 for all the sites indicating high load of HM in the sampling site and progressive deterioration. However, sampling site IS1, IS2 and IS3 are very close to each other (shown in Figure 1), showed comparatively lower PLI value than the other sites which may be due to the lower concentration of elements found in those sites. Prosad et al. [55] reported a low-level PLI value (PLI < 1) for the heavy metals Cr, Cd, Cu, Ni, As and Pb in the soil samples of different areas of Kushtia and Jinaidah districts of Bangladesh.

### 3.3. Apportionment of Possible Sources of Soil Pollution

**Figure 3:**
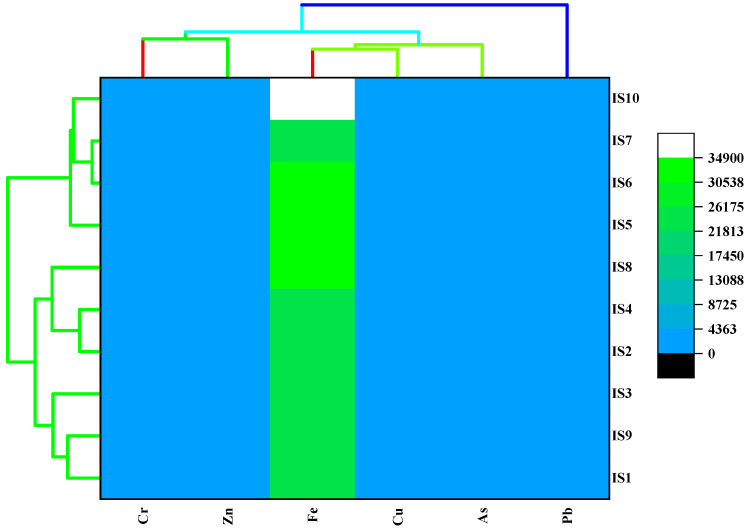
Hierarchical cluster diagram of sites of industrially affected soil samples (the distance reveals the degree of association between different sites based on the dissimilarity of heavy metals concentrations in soil samples).

Cluster analysis is designed for the better identification of a distinguishable group of items at the sampling site against the detected parameters with respect to notable variability [25]. An almostidentical group of sites is presented in a cluster group, and the unalike site is plotted in another cluster group to identify the specific areas to depict the extent of contamination [40]. In the present study, the two-way hierarchical cluster heatmap and dendrogram, developed by the Ward linkage method with Euclidean distance, were prepared, and the result is portrayed in Figure 3. In the vertical portion, the dendrogram provided two clusters: As, Zn, Fe Cu and Cr had been confined in cluster 1, and Pb was displayed in cluster 2, which was mostly confirmed in line with the PCA result. Such findings strongly confirmed a similar origin of the selected metal elements. In contrast, the horizontal dendrogram rendered three clusters, where IS1, IS9 and IS3 were imparted to cluster 1; cluster 2 imparted IS10, IS7, IS6 and IS5 sites, and finally, IS2, IS4 and IS8 were confined to cluster 3.

**Figure 4:**
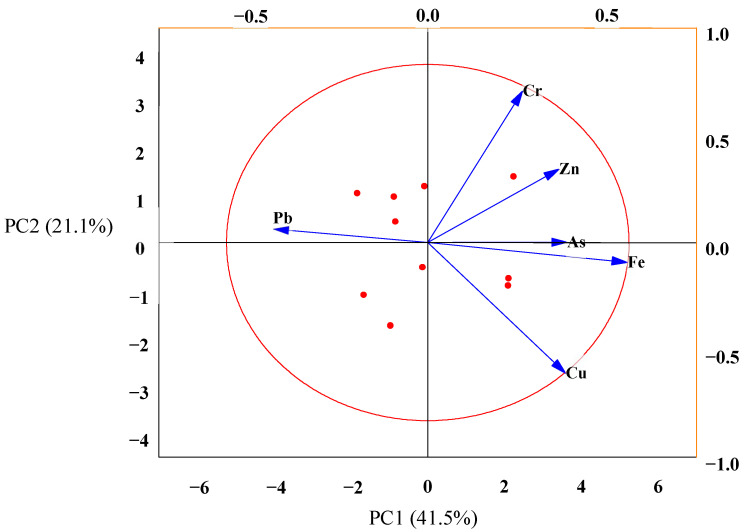
Heavy metals pollution source identification by PCA in the soil samples.

The principal component analysis (PCA) was conducted to determine the correlation and retrospective sources of the tested elements [4,25]. The corresponded PCA was executed following a rotated component plot concerning the loadings depicted in Figure 4. The PCA plot was based on the eigenvalues greater than 1, and the relations were apparent. In Figure 4, all the metal contents moved toward the positive direction of the axis PCA1, which revealed that they were associated, with each other [57]. The executed PCA resulted in two corresponding factors; PC1 contributed 41.5%, while PC2 rendered 21.1% of the total variance. Cr was at 0.8 substantial positive loads, indicating an anthropogenic source of contaminants, and Zn, Pb and As were also positive, but below 0.5 indicated moderate loadings also indicating the anthropogenic source of contaminants. It also indicates that the Zn, Pb and As contaminants come from similar types of industrial activities located in the same grouped sites. In contrast, Fe and Cu were found negative loadings where the Cu value indicates strong loadings (−0.7) reflecting a lithogenic source.

### 3.4. Heavy Metal Contents in Vegetable Samples

The concentration of HMs (Fe, Cr, Cu, Zn, As, Pb) present i−n the examined vegetable samples are illustrated in Table 5. The maximum concentration of Fe was found 368.11 mg/kg in Radish and minimum was found 45.78 mg/kg in Potato. Fe is present in the earth crust abundantly, thus most of the vegetables contain more or less Fe in their tissues. The maximum and the minimum concentration of Cr were found 2.11 and 0.21 mg/kg in Red amaranth and Potato, respectively, which are within the legislative limit of 23.00 mg/kg suggested by WHO [58]. Upto certain amount (200 mg/day) of Cr is acceptable as it is necessary for carbohydrate, fat and cholesterol metabolism but chronic exposure may cause harmful effect on liver and kidney [59]. The maximum and the minimum concentration of Cu were found 19.39 and 7.21 mg/kg in Spinach and Papaya, respectively, which are within WHO [58] suggestive value (Table 5). Usually, sizeable amount of Cu is found in green leafy vegetables and most of the vegetables have some Cr content. However, the Cu levels in the vegetables in the present study were similar with the reported value of vegetables by Adedokun et al. [60]. The maximum and the minimum amount of Zn were found 12.32 and 3.96 mg/kg in Coriander leaf and Carrot, respectively, and Zn is found to be abundant in all the vegetables studied. In a study Jolly et al. [21] reported a higher value of Zn in the vegetable sample collected from Isward, Bangladesh. The maximum and minimum concentration for As were found 5.35 and 2.86 mg/kg in Tomato and Radish, respectively; while for Pb the maximum and the minimum concentration was found 9.41 and 1.67 mg/kg in Potato and Bean, respectively. As and Pb were found to show a value many-fold higher than the WHO [58] recommended value (Table 5).

The high level of Pb and As in the plant species may be explained by the pollutants present in irrigation water, land texture, used fertilizer or due to pollutants from highway traffic and the industrial establishment around the sampling site [61]. However, Pb and As are highly toxic elements, and their dietary intake via vegetables may pose both acute and chronic poisoning and can affect the liver, kidney, vascular tissue, skin and the immune system adversely [62]. It is noticeable that studied HMs are distributed in vegetables in a scattered way, which may be issues of crop species variation, growth period of crops, various metal uptake capabilities of crop plant, and the part used for the edible purpose. Thus, in a study, Tsafe et al. [63] observed a contradictory value of Pb, Cu, Zn, Cr, and Fe as 29.66, 1.13, 68.91, 16.73, and 195.25 mg/kg in different varieties of vegetables grown in Yargalma, Northern Nigeria. However, Adedokun et al. [60] reported a lower value of Cu, Zn, Ni, and a higher value of Cd, Pd Cr than the threshold value suggested by WHO/FAO [58] in some leafy vegetables cultivated in floodplains and farmland of Lagos, Nigeria.

### 3.5. Metal Pollution Index (MPI)

The overall heavy metal pollution in the various studied vegetables is estimated by calculating MPI (Table 5). The highest MPI value was found for Papaya (14.765) and the lowest for pumpkin (4.782), and both belong to fruit vegetables. However, leafy vegetables such asspinach (8.120), cabbage (7.905), red amaranth (11.713) and coriander (9.588) pose a comparatively high MPI value, which was in agreement with the findings of Kashem and Singh [64]. Ahmed and Goni [65] also reported that leafy vegetable accumulates the highest level of heavy metals. Song et al. [66] believed the ability of leafy vegetables to transfer metals from soil in different parts of the plant is higher than that of fruit vegetables. However, in this study, no particular trend was observed for leafy or non-leafy vegetables and hence, the variation of MPI value can be explained by variable uptake capacity of HMs by the plant, morphology and physiology, exclusion, accumulation and retention, etc. Furthermore, MPI values for all the vegetables were estimated relatively high and can be attributed to the presence of a high level of heavy metal in the soil, and suggested avoiding consumption.

### 3.6. Bioaccumulation Factor (BCFs)

The transfer of HMs from soil to plant (BCFs) depends on the soil physicochemical characteristics; types of HM accumulation and plant species [67]. Heavy metal transfer from soil to crops causes many agronomic, environmental and human health problems [68,69,70]. Many researchers have reported that many plant species can tolerate and bio-accumulate high levels of heavy metals in their tissues [71,72]. Likewise, Lettuce (Lactucasativa), a leafy vegetable popularly consumed by humans, accumulates high concentrations of Zn, Cu, Cd, Cr, La, Fe, Ni, Mn, Pb, Ti, Sc and V [73]. In this study, bioaccumulation factors (BCFs) of six heavy metals (Fe, Cr, Cu, Zn, As Pb) from soil to edible portion of different vegetables are calculated and obtained results are computed in Table 6, which revealed BCF values varied considerably in different species of vegetables. Comparatively, a higher BCF value is found for Cu, Zn, As and Pb and hence the ranges are 0.4577–0.1702, 0.2475–0.0796, 0.1581–0.1019, 0.6874–0.1220, respectively. Sultana et al. [74] reported that a BCF value of 0.1 is the indication of excluding elements from their tissues and when the BCF value is more than 0.2, there is a great possibility for metal contamination of vegetables by anthropogenic sources. It is worth mentioning that BCF value for As in Spinach, Cabbage, Red Amaranth, Tomato and Radish are comparatively higher than other vegetables and can be considered as arsenic (As) extractor while, Coriander, Brinjal, Bean, Pumpkin, Bottle gourd, Cauliflower, Radish and Potato are Lead (Pb) extractor (Table 6). However, BCF values of Cu and Zn range from 0.1702–0.4577 and 0.079–0.2475, respectively, but all the vegetables showed very low BCFs values for Fe (0.0135–0.0017) and Cr (0.0344–0.0034), indicating less effective translocation capacity. Nevertheless, all the studied vegetables had a BCF value < 1, indicating, the accumulation of heavy metals (Fe, Cr, Cu, Zn, As, Pb) by the plants’ species is relatively low and less effectively translocate from soil to the edible portion of the vegetables [23].

### 3.7. Impact of HMs Contaminated Soil on Human Health

The adverse effect of HMs contaminated soil on human health (carcinogenic and non-carcinogenic) through ingestion, inhalation and dermal contact and health risk (carcinogenic and non-carcinogenic) due to consumption of HMs contaminated vegetables for both adults and children are calculated and computed in Table 7.

#### 3.7.1. Soil to Human Risk Assessment

In this study, health risks due to direct soil exposure are calculated considering average metal concentrations (Fe, Cr, Cu, Zn, As, Pb) of affected soil in the ten sampling sites and computed in Table 7. In case of ingestion route the highest HQ value was found for As (adult: 6.59 × 10^−^^2^; child: 6.15 × 10^−^^1^) and lowest for Zn (adult: 1.7 × 10^−^^4^; child: 1.09 × 10^−^^3^). In contrast, for the path inhalation, maximum HQ value was found for Cr (adult: 2.27 × 10^−^^4^; child: 3.78 × 10^−^^4^) and minimum for Zn (adult: 1.72 × 10^−^^8^; child: 2.89 × 10^−^^8^), while for dermal contact maximum HQ value was found for As (adult: 6.72 × 10^−^^3^; child: 4.66 × 10^−^^2^) and minimum for Zn (adult: 8.01 × 10^−^^7^; child: 5.74 × 10^−^^6^), respectively. However, the possible non-carcinogenic risk effect of HMs contaminated soil exposure (HQ_soil_) through all three paths can be ranked in the order of As > Fe > Cr > Pb > Cu > Zn for adults, with a similar trend for the child as well, but in each case, the estimated value was found higher in children compared to adult (Table 7), which can be attributed by higher respiration rates per unit body weight, unawareness, unconscious hand-to-mouth activities with contaminated soils, and immature detoxification capabilities of children [75,76]. Nevertheless, HQ_soil_ for all the calculated elements were found <1 for both adult and child (Table 7) indicating low risk in the study area, hence a similar trend was reported by Prosad et al. [55] in the soil samples collected from Jhenidah and Kushtia districts of Bangladesh. However, it is noticeable that the ingestion pathway dominated the dermal and inhalation pathway, and the results are in good agreement with the findings of [10,11,40,76]. The lifetime cancer risk (CR) for the carcinogenic metals Cr, As, Pb IARC [77] has been calculated for all three paths (ingestion, inhalation, dermal contact) and the respective CR values are summarised in Table 7. Calculated CR value (Table 7) for heavy metal Cr was found 2.69 × 10^−^^7^ and 9.74 × 10^−^^6^ for adults and children, respectively, which is level I contamination for adults, indicating extremely low risk and completely acceptable, whereas for children the contamination level is II, which is low in risk and suggested not to eager about the probable risk [44,45]. Furthermore, the CR value for Arsenic (As) was found 2.07 × 10^−^^5^ for adults, which is a level III contamination, indicating low-medium risk but not too mindful of the risk and the CR value for As in children was found 3.00 × 10^−^^4^, a level V contamination, indicating medium-high risk and suggested to care about the risk and to take necessary action [44,45]. On the other hand, CR value for Pb is 8.68 × 10^−^^8^ and 8.10 × 10^−^^7^ for adults and children, respectively, which was in the Level-I category, indicating extremely low risk and lies within the acceptable range [44,45]. In a study, Rahman et al. (2019) found cancer risk levels for Cr and As in the range of 2.97 × 10^−^^6^ to 5.49 × 10^−^^6^ and 5.61 × 10^−^^7^ to 1.28 × 10^−^^6^, respectively, in the soil dust sample of Dhaka city. A lower CR value was also reported by Kormoker et al. [52] for children and adults for the industrially affected agricultural soil of different areas of Jinaidah and Kushtia of Bangladesh. Furthermore, Rahman et al. [40] estimated CR value in soil samples of different schools in Dhaka, Bangladesh and found 1.41 × 10^−^^9^ and 4.323 × 10^−^^9^ for adults and children, respectively. However, lifetime cancer risk (CR) is found higher in children than adults in each case, which is consistent with the finding by Proshad et al. [55], where the calculated CR values were 9.96 × 10^−^^4^ and 1.81 × 10^−^^5^ for As and Pb, respectively, for the child, while those for adults were 4.16 × 10^−^^4^ and 4.50 × 10^−^^6^, respectively, in the agricultural soil of Jhinaidhah and Kushtia district of Bangladesh.

#### 3.7.2. Soil to Vegetable to Human Risk Assessment

In general, a variety of vegetables are consumed by different population segments throughout the year. Thus, estimation of the average intake of metal from the different varieties of vegetables is more realistic, therefore, the mean concentration of metals (Fe, Cr, Cu, Zn, As, Pb) in the 15 varieties of vegetables are considered for the calculation of health risk indices (EDI, THQ and CR) in this study, which are computed in Table 7 for both the population group (adults and child). The trend for estimated daily intake of metal (EDI) from consumption of vegetables are Fe(1.52 × 10^−^^4^) > Cu(1.24 × 10^−^^5^) > Zn(9.80 × 10^−^^6^) > Pb(3.42 × 10^−^^6^) > As(1.70 × 10^−^^6^) > Cr(6.70 × 10^−^^7^) and Fe(2.39 × 10^−^^4^)> As(2.70 × 10^−^^5^) > Cu(1.95 × 10^−^^5^)> Zn(1.40 × 10^−^^5^) > Pb(5.00 × 10^−^^6^) > Cr(1.00 × 10^−^^6^) for adult and child (Table 7), respectively. The EDI of heavy metals via dietary intake of vegetables grown around Pb/Zn smelter of southwest China among different population groups was found in the decreasing order of Zn > Cu > Pb > As [33], which is consistence with the present findings. Calculated THQ value for the studied vegetables for adult and child were found 2.17 × 10^−^^4^, 5.00 × 10^−^^6^, 4.10 × 10^−^^5^, 3.00 × 10^−^^5^, 5.68 × 10^4^, 1.71 × 10^−^^3^ and 3.42 × 10^−^^4^, 8.00 × 10^−^^6^, 6.50 × 10^−^^5^, 4.80 × 10^−^^5^, 8.93 × 10^−^^4^, 2.69 × 10^−^^3^, respectively, and all the values were below the unity (< 1), indicating no potential non-cancer risk from the vegetables upon consumption by both the population group. However, it is mention-worthy that, in each case, the THQ values for children are higher than the adult. This scenario is also consistent with the findings of [78]. In a previous study, Jolly et al. [21] reported to found THQ values for Fe, Cu, Cr, Pb, and Zn as 0.462, 0.512, 0.0003, 0.767 and 1.558, respectively, from the vegetable samples collected from Rooppur, Pabna, Bangladesh, which were much higher than the present value. Measured CR value for the carcinogenic element Cr, As, Pb was found 2.01 × 10^−^^9^, 2.55 × 10^−^^8^, 2.92 × 10^−^^8^ for adult and 3.16 × 10^−^^9^, 4.02 × 10^−^^8^, 4.58 × 10^−^^8^ for child, respectively. All the CR values are below the threshold limit of > 1 × 10^−^^6^ and according to Li et al. [44], CR values lie in the Level-I category in an extremely low-risk zone and are acceptable. Similar findings were reported by Urrutia-Goyes et al. [79] and Bourliva et al. [80] in the vegetable samples of the contaminated area. In contrast, Proshad et al. [55] reported that crops grown in Jhinadah and Kushtia district, Bangladesh, are polluted with Cd, As, and Pb and pose lifetime carcinogenic risks for both populations.

#### 3.7.3. Comparison of Contamination Pathway

**Figure 5:**
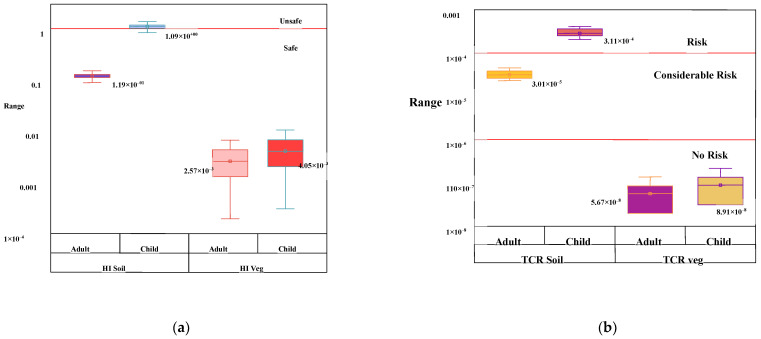
(**a**). Box-whisker plot showing Hazard Index for the assessment of non-carcinogenic risk by the studied heavy metal contaminated soil in adults and child direct and indirect path way. (**b**). Box-whisker plot showing total carcinogenic risk by the carcinogen present in the soil in adults and child by direct soil exposure and via vegetable consumption.

A comparison between soil-human and soil-vegetable-human exposure pathways was made to evaluate the most vulnerable path of heavy metal contamination for the human body. Figure 5a illustrated the non-carcinogenic health risk accounting by direct soil exposure and vegetable consumption via the calculated Hazard Index of both the exposure route. The mean value of total health risk, HI for soil was measured at 1.19 × 10^−^^1^ and 1.09 for adults and children, respectively. Lemly [75] categorised HI value as <0.1 are negligible, 0.1 < HI < 1 pose low significant health effect, 1 < HI < 4 pose medium significant health effect and HI > 4 pose a very high risk; thus, HI for adult lied 0.1 < HI < 1, indicating low significant health effect, while for the child, HI > 1 indicating medium significant health risk. In contrast, HI, accounting for vegetable consumption, was measured at 2.57 × 10^−^^3^ and 4.05 × 10^−^^3^ for adults and children, respectively, appraising HI < 1 revealing no risk. Similarly, total lifetime carcinogenic risk value (TCR) for soil and vegetable for both the population groups were measured (Figure 5b) and for direct soil, exposure was found 3.01 × 10^−^^5^ and 3.11 × 10^−^^4^ for adults and children, respectively, indicating low to medium risk for adult and medium to high risk for children, [44,45]. However, the TCR value derived for vegetable consumption was measured at 5.67 × 10^−^^8^ and 8.91 × 10^−^^8^ for adults and children, respectively, which lied in the Level-I category and posed an extremely low risk. The overall result ascertains that soil to the human path is more hazardous than the soil-vegetable-human path.

## 4. Conclusions

This study has assessed heavy metal contamination in the soil of agricultural land adjacent to an industrial zone of Ashulia, Savar, Bangladesh and their accumulation in the cultivated vegetables on that field. The elevated level of HMs (Fe, Cr, Cu, Zn, As, Pb) were found in the industrially affected soil compared to control soil and the estimated value of EF, I_geo_ and CF supported this result. Moreover, the calculated PLI value showed a value greater than unity for all the soil samples, indicating decreasing of soil quality and increase of heavy metal pollution in the entire site. Multivariate statistical analysis ascertains that Fe, Cu, and Zn have lithogenic sources, whereas Cr, As, and Pb come from anthropogenic activities. However, the concentration of all the measured HMs in vegetables found within the legislative value suggested by FAO/WHO except for As and Pb. Comparatively, a high level of MPI value was measured in all the vegetables and can be ranked as PA > BR > RA > BE > CO > RD > SP > CA > TO > PO > CF > GB > BG > CA > PP. Calculated BCF values showed lower than unity for all the elements indicating low HMs uptake capacity by the plant; however, BCF values are found near to unity by Potato (0.6874), Radish (0.5435), Coriander (0.5157), Brinjal (0.4711) and Cauliflower (0.3156) for Pb, indicating metal contamination by anthropogenic activities and suggested regular monitoring. Estimated HQ via direct soil exposure can be ranked as HQ_ing_ > HQ_derm_ > HQ_inhel_ regardless of age, and HQ values for all the elements in the entire three pathways for adults and children were <1, indicating not to pose any health effect. Similarly, HQ via vegetable consumption was found below unity for both the population group and recommended safety limit. Nonetheless, HI value via direct soil exposure was measured <1 for adults and >1 for the child, on the other hand, total lifetime carcinogenic health risk for adults lied within level II (1 × 10^−^^5^ to 1 × 10^−^^6^) but in Level-V (1 × 10^−^^4^ to 5 × 10^−^^4^) for children, stipulated medium to high risk. In contrast, HI and TCR values for the population group via dietary intake of vegetables collected from the industrially affected soil site found within the safety limit recommended by international bodies.

## Figures and Tables

**Table 1:** Description of the health risk indices utilized to estimate heavy metal impact from soil to human.

Indices	Soil to Human			
	Purposes	Equation and Description		References
ADD_ing_	Ingestion of heavy metals through soil/dust	${\mathrm{ADD}}_{\mathrm{ing}}=C\times \frac{IngRXEFXED}{BWXAT}\times 10$ ^−6^	(9)	[36,37,40]
		where, ADD_ing_ = average daily intake of heavy metals, ingested from the soil, mg/kg-day, C = concentration of heavy metal mg/kg; IngR is = soil ingestion rate (200 for child and 100 for adult, mg/day); EF= exposure frequency (180 days/year); ED = exposure duration(6 for child and 24 for adult, years); BW = body weight (15 for child and 70 kg for adult); AT = time period (365 × ED for child and 365 × ED for adult, days); CF= conversion factor (10^−6^, kg/mg).		
ADD_inh_	Inhalation of heavy metals via soil particulates	${\mathrm{ADD}}_{\mathrm{inh}}=C\times \frac{IngRXEFXED}{PEF\times BWXAT}$	(10)	[36,37,40]
		where, ADDinh = intake of heavy metals, inhaled from the soil, mg/kg-day, C = concentration of heavy metal, mg/kg; IngR = soil inhalation rate (7.6 for child and 20 for adult, mg·day^−1^); PEF= particulate emission factor (1.36 × 10^9^m^3^/kg); EF, ED, BW, and AT are as defined earlier in Equation (10).		
ADD_dermal_	Dermal contact with soil via skin	${\mathrm{ADD}}_{\mathrm{dermal}}=C\times \frac{SAXAFXABS\times EF\times ED\;}{BWXAT}\;$× 10^−6^	(11)	[36,37,40]
		where, ADD_dermal_ = exposure dose via dermal contact, mg/kg/day; C= concentration of heavy metal in soil, mg/kg, SA= exposed skin area (1150 for child and 2145 for adult, cm^2^); AF = adherence factor (0.2 for child and 0.07 for adult, mg.cm^−2^ day^−1^); ABS= fraction of the applied dose absorbed across the skin (0.001) for all element but for Arsenic, ABS = 0.03. EF, ED, BW, and AT are as defined earlier in Equation (10)		
HQ_soil_	Non-carcinogenic health risk	${\mathrm{HQ}}_{\mathrm{soil}}=\frac{\mathrm{ADD}\;\left({\mathrm{ADD}}_{\mathrm{inges}}\;+\;{\mathrm{ADD}}_{\mathrm{inha}}\;+\;{\mathrm{ADD}}_{\mathrm{derm}}\;\right)}{\mathrm{RfD}}$	(12)	[40]
HI	Hazard Index	HI = ∑HQ_i_ = HQ_ing_ + HQ_inh_ + HQ_der_	(13)	[40]
LAAD	Lifetime average daily dose	${\mathrm{LADD}}_{\mathrm{ing}}=C\times \frac{IngRXEFXED}{BWXAT}\times 10$ ^−6^	(14)	[36,37,40]
		${\mathrm{LADD}}_{\mathrm{inh}}=C\times \frac{IngRXEFXED}{PEF\times BWXAT}$	(15)	
		${\mathrm{LADD}}_{\mathrm{dermal}}=C\times \frac{SAXSLXABS\times EF\times ED\;}{BWXAT}\;$× 10^−6^	(16)	
		All the values are similar as in case of non-carcinogenic risk calculation (Equations (9)–(11)) only AT = 365 × 70 year.		
CR	Lifetime cancer risk for an individual from the average contribution for individual heavy metal for all the path	CR=LAAd×SF	(17)	
		LAAD = Risk_ingestion_ + Risk_inhalation_ + Risk_dermal_	(18)	[40]
TCR	Total excess lifetime cancer risk for an individual accounting for all the carcinogenic metals	${\mathrm{Risk}}_{\mathrm{Total}}\;(\mathrm{TCR})=\;\sum CR$	(19)	[40]

**Table 2:** Description of the health risk indices utilized to estimate heavy metal impact from soil–vegetable- human.

Indices	Vegetable to Human			
	Purposes	Equation and Description		References
EDI	To estimate exposure to heavy metals via vegetable consumption (mg/kg·day)	$EDI=\frac{Cveg\;\times \;IR\;\times \;ED\;\times \;EF}{Bwt\;\times \;AT}\times 10^{-3}$	(20)	[18,36,37]
		where, *Cveg* is the concentrations of heavy metal estimated (mg/kg dry-wt); *IR* is the daily ingestion rate of vegetable adopted by Food contaminated survey Bangladesh (0.089 kg//day for adults and 0.03 kg/day for children considering children consume 1/3 rd of vegetable comparing adult); *EF* is the exposure frequency = 365 days; *ED* is the exposure duration = 65 years; *BW* is the body wt (70 kg for adults and 15 kg for children); *At* is the average exposure time for non carcinogenic effect (*ED* × 365)		
THQ	To assess the non-carcinogenic risks of individual heavy metals via consumption of contaminated vegetables.	$\mathrm{THQs}=\frac{EDI}{RfD}$	(21)	[20,36,37,46]
		where, *ED* = Estimated daily intake of heavy metal via vegetable intake. *RfD* = oral reference dose (mg/person/day) of metals viz. Fe (0.7), Cr (0.14), Cu (0.3), Zn (0.3), As (0.003), Pb (0.002), respectively. THQ < 1 refers non-significant risk effects.		
HI	To estimate the potential non-carcinogenic risk from multiple heavy metals.	$\mathrm{HI}=\sum_{i=k}^{n}THQ$	(22)	[15,20,36,37]
		where, HI is the summation of THQ of the studied element in each vegetable samples and HI > 1 refers significant non-carcinogenic health risk.		
CR	To evaluate the incremental probability of cancer in an individual, over a lifetime, due to exposure to a substantial carcinogen.	CR=EDI×SF	(23)	[20,36,37,46]
		$EDI=\frac{Cveg\times IR\times ED\times EF}{Bwt\times AT}\;\times 10^{-3}$	(24)	
		where, CSF = oral slope factor of carcinogens (mg/kg/day). In the present study only Pb, Cr, As have carcinogenic effect and the SF(slope factor) are 0.0085, 0.003 and 15 × 10^−3^ for Pb, Cr and As respectively. *Cveg*, *IR*, *EF*, *Bwt*, *Ed* values are same as used in Equation (15) and At = 70 × 365.		
TCR	To estimate total excess lifetime cancer risk for an individual	$TCR=\sum CR_{Cr}+{\mathrm{CR}}_{\mathrm{As}}+{\mathrm{CR}}_{\mathrm{Pb}}$	(25)	[46]

**Table 3:** Heavy metal concentration (mg/kg) data for industrially affected soil (n = 3) and control soil (n = 3).

Elements	Sample ID										Mean mg/kg	Maximum mg/kg	Minimum mg/kg	Control Soil ^a^ mg/kg
	IS1	IS2	IS3	IS4	IS5	IS6	IS7	IS8	IS9	IS 10				
Cr	51.18 ± 0.26	62.23 ± 0.28	65.27 ± 0.18	67.23 ± 0.22	57.17 ± 0.21	59.24 ± 0.25	52.18 ± 0.17	68.19 ± 0.24	64.23 ± 0.23	65.77 ± 0.22	61.27	68.19	51.18	13.45 ± 5.78
Fe	22,860 ± 105	24,270 ± 118	23,780 ± 124	22,680 ± 108	32,040 ± 119	33,840 ± 113	21,840 ± 110	32,070 ± 109	24,460 ± 112	34,900 ± 118	27,274	34,900	21,840	21,570 ± 3946
Cu	38.85 ± 0.15	32.24 ± 0.11	40.67 ± 0.13	39.51 ± 0.11	41.78 ± 0.19	51.78 ± 0.11	48.95 ± 0.10	39.66 ± 0.18	40.06 ± 0.19	50.10 ± 0.14	42.36	51.78	32.24	32.43 ± 2.30
Zn	44.88 ± 1.02	46.41 ± 0.97	46.63 ± 0.99	47.14 ± 1.18	49.04 ± 1.15	56.63 ± 0.97	46.69 ± 0.89	56.92 ± 1.05	57.94 ± 0.82	45.44 ± 0.93	49.77	57.94	44.88	35.33 ± 3.50
As	21.13 ± 0.32	25.40 ± 0.29	18.19 ± 0.22	31.43 ± 0.37	25.77 ± 0.23	28.91 ± 0.19	33.22 ± 0.31	37.34 ± 0.24	25.21 ± 0.19	34.17 ± 0.23	28.08	37.34	18.19	6.03 ± 1.81
Pb	11.35 ± 0.17	18.34 ± 0.10	10.78 ± 0.09	18.25 ± 0.12	15.76 ± 0.11	10.51 ± 0.08	18.53 ± 0.17	9.22 ± 0.05	15.11 ± 0.06	9.03 ± 0.16	13.69	18.53	9.03	5.61 ± 2.64

^a^ = mean of five stations, considered as the background data.

**Table 4:** Assessment of degree of pollution by the heavy metal in soil sample.

Sample ID	Element						Assessment
	Cr	Fe	Cu	Zn	As	Pb	
	Enrichment Factor (EF)						
IS1	3.590	1	1.130	1.199	3.306	1.909	The sampling site is minimum enriched by Cu, and Zn; while moderate enrichment was observed for Cr, As and Pb.
IS2	4.112	1	0.884	1.167	3.745	2.905	
IS3	4.402	1	1.138	1.197	2.736	1.743	
IS4	4.754	1	1.159	1.269	4.957	3.094	
IS5	2.862	1	0.867	0.934	2.877	1.891	
IS6	2.807	1	1.018	1.022	3.056	1.194	
IS7	3.832	1	1.491	1.305	1.646	3.262	
IS8	3.410	1	0.823	1.084	4.165	1.105	
IS9	4.211	1	1.089	1.446	3.687	2.375	
IS10	3.022	1	0.955	0.795	3.502	0.995	
Mean	3.700	1	1.055	1.142	3.368	2.047	
	Geo-accumulation Index (Igeo)						
IS1	0.404	−0.151	−0.098	−0.072	0.368	0.130	The site is unpolluted by Fe, Cu and Zn indicating cluster metal; while moderately polluted by Cr, As and Pb indicated anthropogenic source.
IS2	0.489	−0.125	−0.177	−0.058	0.448	0.338	
IS3	0.510	−0.134	−0.078	−0.056	0.303	0.108	
IS4	0.523	−0.154	−0.090	−0.051	0.541	0.336	
IS5	0.452	−0.004	−0.066	−0.034	0.455	0.273	
IS6	0.468	0.019	0.027	0.029	0.505	0.097	
IS7	0.413	−0.171	0.003	−0.056	0.565	0.343	
IS8	0.529	−0.004	−0.089	0.031	0.616	0.040	
IS9	0.503	−0.121	−0.084	0.039	0.445	0.254	
IS10	0.513	0.033	0.013	−0.067	0.577	0.031	
Mean	0.480	−0.081	−0.064	−0.030	0.482	0.195	
	Contamination Factor (CF)						
IS1	3.805	1.060	1.198	1.270	3.504	2.023	The site is moderately contaminated by Fe, Cu, Zn and Pb, while considerable contamination was accounted by Cr and As.
IS2	4.627	1.125	0.994	1.314	4.212	3.269	
IS3	4.853	1.102	1.254	1.320	3.017	1.922	
IS4	4.999	1.051	1.218	1.334	5.212	3.253	
IS5	4.251	1.485	1.288	1.388	4.274	2.809	
IS6	4.404	1.569	1.597	1.603	4.795	1.873	
IS7	3.880	1.013	1.509	1.322	5.509	3.303	
IS8	5.070	1.487	1.223	1.611	6.192	1.643	
IS9	4.775	1.134	1.235	1.640	4.181	2.693	
IS10	4.890	1.618	1.545	1.286	5.667	1.610	
Mean	4.555	1.264	1.306	1.409	4.656	2.440	

**Table 5:** Elemental concentrations in vegetable samples and estimated metal pollution index (MPI) value.

Sample ID	Scientific Name	Edible Part	Element, mg/kg						MPI
			Fe	Cr	Cu	Zn	As	Pb	
Spinach (SP)	*Spinacea oleracea*	Leaf	55.36 ± 1.66	1.05 ± 0.09	19.39 ± 1.99	8.20 ± 0.29	3.82 ± 0.05	<0.12	8.120
Cabbage (CAB)	*Brassica oleracea var. capitata*		133.08 ± 2.09	0.88 ± 0.05	10.48 ± 0.19	6.95 ± 0.11	3.62 ± 0.03	<0.12	7.905
Red Amaranth (RA)	*Amaranathus gangeticus*		277.69 ± 2.31	2.11 ± 0.15	7.89 ± 0.11	10.74 ± 0.23	4.44 ± 0.05	<0.12	11.713
Coriander leaf (CO)	*Cariandum sativum*		145.32 ± 1.91	0.62 ± 0.08	10.34 ± 0.17	12.32 ± 0.18	< 0.01	7.06 ± 0.11	9.588
Tomato (TO)	*Solanum iycopersicum*	Fruit	113.60 ± 1.07	0.72 ± 0.11	8.13 ± 0.08	6.25 ± 0.09	5.35 ± 0.08	<0.12	7.403
Brinjal (BR)	*Solanum melongrna*		66.72 ± 0.56	<0.05	10.84 ± 0.12	7.42 ± 0.10	<0.01	6.45 ± 0.09	13.639
Bean (BE)	*Phaseolus lunatus*		84.11 ± 0.36	<0.05	9.84 ± 0.09	6.83 ± 0.05	<0.01	1.67 ± 0.04	9.857
Pumpkin (PP)	*Cucurbita mochata*		64.23 ± 0.54	0.32 ± 0.04	8.82 ± 0.08	7.30 ± 0.09	<0.01	1.89 ± 0.03	4.782
Bottle gourd (BG)	*Lagenaria siceraria*		80.14 ± 0.41	0.29 ± 0.04	11.13 ± 0.11	7.65 ± 0.08	<0.01	2.14 ± 0.10	5.313
Papaya(PA)	*Carica papaya*		76.65 ± 0.33	<0.05	7.21 ± 0.12	5.83 ± 0.06	<0.01	<0.12	14.765
Green banana (GB)	*Musa acuminata*		77.72 ± 0.31	0.26 ± 0.03	7.24 ± 0.08	5.83 ± 0.09	<0.01	<0.12	5.404
Cauliflower (CF)	*Brassica oleracea var. botrytis*	Inflorescence	119.95 ± 0.98	0.38 ± 0.05	7.62 ± 0.08	5.75 ± 0.06	<0.01	4.32 ± 0.12	6.126
Carrot (CAR)	*Daucus carota var. sativus*	Root	85.35 ± 0.59	0.21 ± 0.02	9.44 ± 0.05	3.96 ± 0.05	<0.01	<0.12	5.087
Radish (RD)	*Raphanus sativus*		368.11 ± 2.11	0.85 ± 0.06	7.35 ± 0.09	6.59 ± 0.07	2.86 ± 0.05	7.44 ± 0.08	8.280
Potato (PO)	*Solanum tuberosum*	Tuber	45.78 ± 0.28	0.21 ± 0.05	10.75 ± 0.13	5.50 ± 0.07	<0.01	9.41 ± 0.14	6.567
Mean			119.59	0.53	9.76	7.14	1.34	2.69	
Max			277.69	2.11	19.39	12.32	5.35	9.41	
Min			45.78	0.21	7.21	3.96	2.86	1.67	
^a^ FAO/WHO,s MPL [58]			-	23.00	40.00	-	0.10	0.10	

^a^ The maximum permissible limit recommended by the Food and Agriculture Organization and the World Health Organization [58].

**Table 6:** Estimated value of Bioaccumulation factor of heavy metals (HMs) from soil to edible part of the vegetable samples.

Sample Id	Bioaccumulation Factor (BCFs)					
	Fe	Cr	Cu	Zn	As	Pb
SP	0.0020	0.0171	0.4577	0.1648	0.1360	0
CAB	0.0049	0.0144	0.2474	0.1396	0.1289	0
RA	0.0102	0.0344	0.1863	0.2158	0.1581	0
CO	0.0053	0.0101	0.2441	0.2475	0	0.5157
TO	0.0042	0.0118	0.1919	0.1256	0.1905	0
BR	0.0024	0	0.2559	0.1491	0	0.4711
BE	0.0031	0	0.2323	0.1372	0	0.1220
PP	0.0024	0.0052	0.2082	0.1467	0	0.1381
BG	0.0029	0.0047	0.2627	0.1537	0	0.1563
PA	0.0028	0	0.1702	0.1171	0	0
GB	0.0028	0.0042	0.1709	0.1171	0	0
CF	0.0044	0.0062	0.1799	0.1155	0	0.3156
CAR	0.0031	0.0034	0.2229	0.0796	0	0
RD	0.0135	0.0139	0.1735	0.1324	0.1019	0.5435
PO	0.0017	0.0034	0.2538	0.1105	0	0.6874

**Table 7:** Health risk assessment value from soil-human and soil-vegetable-human pathway.

Risk Indices	Population	Element					
		Cr	Fe	Cu	Zn	As	Pb
		Soil-Human					
HQing	Adult	1.44 × 10^−2^	2.74 × 10^−2^	7.46 × 10^−4^	1.17 × 10^−4^	6.59 × 10^−2^	2.76 × 10^−3^
	Child	1.34 × 10^−1^	2.56 × 10^−1^	6.96 × 10^−3^	1.09 × 10^−3^	6.15 × 10^−1^	2.57 × 10^−2^
HQinh	Adult	2.27 × 10^−4^		1.09 × 10^−7^	1.72 × 10^−8^	9.66 × 10^−6^	4.03 × 10^−7^
	Child	3.78 × 10^−4^		1.81 × 10^−7^	2.89 × 10^−8^	1.60 × 10^−5^	6.88 × 10^−7^
HQderm	Adult	9.93 × 10^−4^		3.40 × 10^−6^	8.01 × 10^−7^	6.72 × 10^−3^	2.54 × 10^−5^
	Child	6.96 × 10^−3^		2.45 × 10^−5^	5.74 × 10^−6^	4.66 × 10^−2^	1.73 × 10^−4^
HQ soil	Adult	1.56 × 10^−2^	2.74 × 10^−2^	7.50 × 10^−4^	1.18 × 10^−4^	7.27 × 10^−2^	2.78 × 10^−3^
	Child	1.42 × 10^−1^	2.56 × 10^−1^	6.99 × 10^−3^	1.10 × 10^−3^	6.62 × 10^−1^	2.59 × 10^−2^
CRing	Adult					2.97 × 10^−^^5^	8.68 × 10^−^^8^
	Child					2.77 × 10^−^^4^	8.10 × 10^−^^7^
CRinh	Adult	2.67 × 10^−^^7^				4.39 × 10^−^^8^	
	Child	4.73 × 10^−^^7^				7.79 × 10^−^^8^	
CRderm	Adult	1.85 × 10^−^^9^				4.66 × 10^−^^9^	
	Child	9.27 × 10^−^^6^				2.33 × 10^−^^5^	
CRsoil	Adult	2.69 × 10^−^^7^				2.97 × 10^−^^5^	8.68 × 10^−8^
	Child	9.74 × 10^−^^6^				3.00 × 10^−^^4^	8.10 × 10^−7^
Soil-Plant-Human							
EDI	Adult	6.70 × 10^−7^	1.52 × 10^−4^	1.24 × 10^−5^	9.08 × 10^−6^	1.70 × 10^−6^	3.42 × 10^−6^
	Child	1.10 × 10^−6^	2.39 × 10^−4^	1.95 × 10^−5^	1.43 × 10^−5^	2.70 × 10^−5^	5.40 × 10^−6^
THQ	Adult	5.00 × 10^−6^	2.17 × 10^−4^	4.10 × 10^−5^	3.00 × 10^−5^	5.68 × 10^−4^	1.71 × 10^−3^
	Child	8.00 × 10^−6^	3.42 × 10^−4^	6.50 × 10^−5^	4.80 × 10^−5^	8.93 × 10^−4^	2.69 × 10^−3^
CR	Adult	2.01 × 10^−9^				2.55 × 10^−8^	2.92 × 10^−8^
	Child	3.16 × 10^−9^				4.02 × 10^−8^	4.58 × 10^−8^

## Data Availability

The datasets used and/or analysed during the current study available from the corresponding author on reasonable request.
